# Hypochloremia in Older Adults with Heart Failure: From Pathophysiology to Prognosis

**DOI:** 10.3390/jcm15145750

**Published:** 2026-07-22

**Authors:** Elisa Fabbri, Lorenzo Maestri, Gabriella Cucinotta, Paolo Muratori

**Affiliations:** 1 Department of Medical and Surgical Sciences, University of Bologna, 40126 Bologna, Italy; 2 Division of Internal Medicine, Morgagni-Pierantoni Hospital, 47121 Forlì, Italy; 3 Department for Life Quality Studies, University of Bologna, 47921 Rimini, Italy

**Keywords:** hypochloremia, heart failure, older adults, chloride, prognosis

## Abstract

Hypochloremia has recently emerged as an independent prognostic marker in heart failure (HF), which is especially common in older adults, where it represents a main cause of death and hospitalization. This narrative review of the literature aims to summarize the main evidence about the clinical significance of hypochloremia in older adults with HF. Particularly, hypochloremia results from the interplay among neurohormonal activation, congestion, renal dysfunction, and diuretic therapy. In older adults, age-related decline in renal function, frailty, multimorbidity, and polypharmacy increase susceptibility to chloride disturbances. Overall, the prevalence of hypochloremia in HF in older people was estimated to be about 25–29%. Although studies specifically involving older adults with HF remain limited, available data support the prognostic relevance of hypochloremia in this population. Moreover, recent evidence has identified distinct hypochloremic phenotypes, namely dilutional and depletional forms, characterized by different pathophysiological mechanisms. Emerging chloride-centered therapeutic strategies may be particularly relevant in older patients. In conclusion, hypochloremia is confirmed as an important marker of disease severity and adverse prognosis in older patients with HF. Routine assessment of serum chloride may improve risk stratification and support a more individualized therapeutic approach. Further prospective studies are needed to clarify the role of chloride-guided management strategies and their impact on clinical outcomes in older patients with HF.

## 1. Introduction

Heart Failure (HF) is a complex clinical syndrome caused by structural and/or functional cardiac abnormalities leading to increased intracardiac pressures and/or inadequate cardiac output at rest and/or during exertion [1]. It represents the final common pathway of several cardiovascular diseases and currently affects more than 64 million people worldwide [2], with a major impact on healthcare systems, society, and quality of life. Its prevalence increases exponentially with aging, reaching approximately 20% among individuals older than 75 years [3] and represents a major cause of morbidity, hospitalization, and mortality in older adults [1].

HF is characterized by the activation of multiple neurohormonal pathways, including the renin–angiotensin–aldosterone system and the sympathetic nervous system, as compensatory mechanisms that, however, ultimately have deleterious, worsening effects such as sodium and water retention, renal dysfunction, and electrolyte imbalance [4].

Electrolyte disturbances are common in both acute and chronic HF and result from the interplay between disease pathophysiology and pharmacological treatment, particularly diuretic therapy. These alterations are consistently associated with worse clinical outcomes and poorer prognosis [5].

Traditionally, most attention has focused on sodium abnormalities. In particular, hyponatremia—primarily reflecting dilutional water excess—has long been recognized as a strong negative prognostic marker associated with increased mortality and rehospitalization in HF patients [6,7].

In recent years, however, increasing interest has emerged regarding the role of chloride, which is known to be a key regulator of acid–base balance, plasma osmolality, renal tubular function, and neurohormonal activation [8]. Emerging evidence suggests that hypochloremia is not merely an epiphenomenon of advanced HF or intensive diuretic therapy, but rather an independent marker of disease severity associated with diuretic resistance, enhanced neurohormonal activation, persistent congestion, and adverse clinical outcomes [9,10,11].

Electrolyte disturbances are particularly common in older adults due to the combined effects of age-related physiological changes, reduced renal reserve, multimorbidity, frailty, malnutrition, and polypharmacy. Aging is associated with impaired renal concentrating and diluting capacity, altered tubular handling of sodium and chloride, and reduced adaptive responses to fluid and electrolyte imbalance. In older patients with both acute and chronic HF, these mechanisms are further amplified by congestion, neurohormonal dysregulation, and extensive use of diuretic therapy.

In this framework, the goal of the present narrative review is to comprehensively synthesize the current evidence regarding the prognostic role of hypochloremia in HF, with a special focus on evidence related to older patients. Specifically, this review aims to discuss the pathophysiological mechanisms underlying hypochloremia, its epidemiology, and its prognostic role in older adults with HF.

## 2. Methods

A narrative literature review was conducted by searching the PubMed database (Bethesda, MD, USA) using combinations of the following keywords: “*heart failure*,” “*chloride*,” “*hypochloremia*,” “*diuretic resistance*,” and “*older adults*.” Observational studies, randomized controlled trials (RCTs), systematic reviews, and meta-analyses published from 2015 onward were considered. Earlier studies were also included when they provided essential background information or contributed to key concepts relevant to the topic. Priority was given to studies involving older adults.

More than 200 articles were initially identified and screened. Articles published in languages other than English were excluded. Titles and abstracts were independently reviewed by the authors, and the reference lists of selected articles were manually screened to identify additional relevant studies. Full-text articles were retrieved for all studies considered potentially eligible. In keeping with the narrative nature of this review, the final selection of studies was based on their methodological quality, clinical relevance, and contribution to the objectives of the review.

## 3. Overview of the Chloride Homeostasis

Chloride is the major anion in the extracellular compartment, with a normal plasma concentration between 98 and 106 mEq/L. Chloride homeostasis results from a dynamic balance between intake, distribution, and excretion, regulated primarily by the kidneys, neurohormonal systems, and acid–base status [8].

Chloride is introduced through the diet, mainly as sodium chloride (NaCl), and is efficiently absorbed in the gastrointestinal tract. The kidney represents the principal regulator of plasma chloride concentration, modulating its excretion and reabsorption along different segments of the nephron. Approximately 60–70% of filtered chloride is reabsorbed in the proximal tubule through both paracellular and transcellular pathways, largely driven by sodium and water reabsorption. Additional reabsorption occurs in the thick ascending limb of the loop of Henle via the Na^+^-K^+^-2Cl^−^ cotransporter (NKCC2), in the distal convoluted tubule via the Na^+^-Cl^−^ cotransporter (NCC), and in the collecting duct through chloride/bicarbonate exchangers such as pendrin [11].

Aging is associated with profound structural and functional changes in the kidneys that impair chloride homeostasis and increase susceptibility to hypochloremia. Progressive nephron loss, glomerulosclerosis, interstitial fibrosis, and tubular atrophy are accompanied by a gradual decline in glomerular filtration rate (GFR) and renal functional reserve [12,13,14]. In parallel, the efficiency of tubular transport mechanisms responsible for sodium and chloride reabsorption declines, particularly within the proximal tubule, the thick ascending limb of the loop of Henle, and the distal convoluted tubule [13]. Experimental studies suggest age-related alterations in the expression and activity of tubular transporters involved in sodium and chloride handling, including NKCC2 and NCC [15,16], resulting in impaired renal ability to respond to variations in volume status and acid–base balance. In addition, aging is associated with attenuation of the renin–angiotensin–aldosterone system (RAAS), characterized by reduced renin and aldosterone secretion and diminished renal responsiveness to these hormones. Although circulating cortisol levels tend to increase modestly with age, this rise appears insufficient to compensate fully for the decline in mineralocorticoid activity. Consequently, the blunted neurohormonal response further impairs sodium and chloride reabsorption during hypovolemic states and reduces the kidney’s ability to restore extracellular fluid volume following fluid loss [17].

The aging kidney also exhibits a reduced ability to both concentrate and dilute urine as a consequence of impaired renal medullary osmotic gradient and decreased responsiveness to vasopressin [18]. This defect predisposes older adults not only to dehydration and electrolyte depletion but also to dilutional hypochloremia under conditions of water retention. Collectively, these age-related renal alterations substantially reduce the kidney’s adaptive reserve, making older individuals particularly vulnerable when exposed to physiological or pathological stressors [13].

Consequently, hypochloremia develops more readily in the presence of precipitating conditions frequently encountered in geriatric patients, including gastrointestinal fluid losses, reduced oral intake, diuretic therapy, heart failure, chronic kidney disease, and other disorders affecting fluid and electrolyte balance.

Furthermore, emerging evidence suggests that aging is associated with increased mineralocorticoid receptor expression and activation in extra-renal tissues, particularly in the cardiovascular system, where these changes have been implicated in vascular remodeling, myocardial fibrosis, inflammation, and age-related functional decline [19]. Whether similar alterations occur in the aging kidney or influence renal chloride handling remains unknown. Likewise, age-related changes in intestinal barrier integrity and function may indirectly influence electrolyte homeostasis through inflammatory pathways and impaired intestinal absorptive function [20]. However, the available evidence supporting a direct role of these mechanisms in the development of hypochloremia is limited.

Beyond its role in electrolyte balance, chloride is a key determinant of tubuloglomerular feedback, as it is sensed by the macula densa to regulate renin release and glomerular filtration rate [21]. Reduced tubular chloride delivery stimulates renin secretion and activation of the RAAS, whereas increased chloride delivery suppresses this response. Chloride also plays a central role in acid–base homeostasis through its reciprocal relationship with bicarbonate [11]. In older patients, the age-related decline in renal functional reserve, together with the high prevalence of chronic kidney disease, multimorbidity, frailty, and polypharmacy, may further impair chloride regulation and amplify the clinical consequences of hypochloremia.

## 4. Characteristics of Older Adults with HF and Hypochloremia

**Figure 1:**
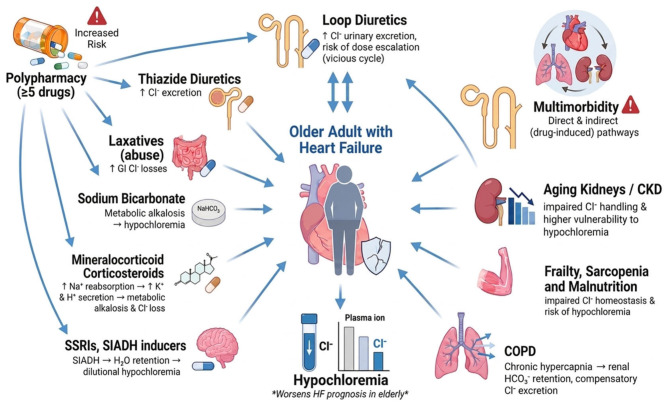
Characteristics of older adults with HF and vulnerability to hypochloremia.

Older adults represent the most vulnerable subgroup among patients with heart failure and are also the population in which hypochloremia occurs more frequently and carries a worse prognosis [22]. The prevalence of hypochloremia in this setting is consistently higher than in younger cohorts [23] and is associated with poorer outcomes due to the combined effects of pathophysiological alterations, progressive decline in renal function, polypharmacy, and the coexistence of comorbidities that affect chloride homeostasis, as summarized in Figure 1.

### 4.1. Frailty, Sarcopenia, and Malnutrition

Frailty, sarcopenia, and malnutrition are highly prevalent among older adults with HF and are associated with increased hospitalization, disability, and mortality in this population [24,25,26]. Several mechanisms may link these geriatric syndromes to chloride depletion. Malnutrition is frequently associated with reduced nutritional intake and impaired physiological reserve, potentially contributing to disturbances in fluid and electrolyte homeostasis [27]. In addition, frail patients present increased vulnerability to stressors, impaired adaptive responses to acute illness, an increased risk of dehydration and inadequate oral intake, and greater susceptibility to adverse drug reactions, particularly those related to diuretic therapy and polypharmacy [28,29]. Sarcopenia is associated with profound changes in body composition and fluid distribution that may increase vulnerability to disturbances in fluid and electrolyte homeostasis [30].

### 4.2. Multimorbidity

One of the main characteristics of aging individuals is the accumulation of multimorbidity, defined as the coexistence of two or more chronic conditions in the same individuals [31]. Notably, multimorbidity may amplify the risk of hypochloremia through multiple interacting mechanisms. Chronic kidney disease, chronic obstructive pulmonary disease, diabetes, atrial fibrillation, and malnutrition frequently coexist in older adults with HF and contribute to a complex clinical milieu that may predispose to electrolyte disturbances, including hypochloremia, either through disease-related mechanisms or through the therapies required for their management.

### 4.3. Renal Dysfunction

Declining renal function is a hallmark of physiological aging [32,33]. This process is associated with structural changes in the renal parenchyma, including reduction in functional glomerular mass, interstitial fibrosis, and nephron loss, all contributing to overall deterioration of renal function [12]. The decline in GFR is accompanied by alterations in tubular electrolyte regulation, including chloride handling [34]. As already pointed out, animal models have demonstrated reduced expression of NKCC2 and NCC in older organisms, impairing the kidney’s ability to adapt to changes in acid–base balance and fluid-electrolyte homeostasis [15]. This impaired tubular regulation is also evident in chronic kidney disease (CKD), where the progressive reduction in functional nephron mass increases susceptibility to electrolyte disturbances, especially in the presence of precipitating factors such as loop diuretic therapy, recurrent vomiting, or reduced dietary intake [35].

### 4.4. Chronic Obstructive Pulmonary Disease (COPD)

Chronic obstructive pulmonary disease (COPD) is a frequent comorbidity in patients with HF, with a prevalence of approximately 13% in this population [36], and is particularly common among older adults [37]. In advanced COPD, chronic hypercapnia induces renal metabolic compensation characterized by increased tubular bicarbonate reabsorption, leading to elevated serum bicarbonate levels. To maintain electroneutrality, chloride is consequently excreted, favoring the development of hypochloremia. Moreover, chronic hypercapnia and acute COPD exacerbations may adversely affect cardiac function, worsening heart failure status [38] and increasing the use of loop diuretics, which further enhance urinary chloride loss. Hypochloremia itself has also been independently associated with a higher risk of COPD exacerbations [39], accelerating the previously described vicious cycle.

### 4.5. Polypharmacy

Polypharmacy (the concomitant use of five or more medications) is linked to multimorbidity and is extremely common among older patients with heart failure, reaching rates as high as 95% at discharge following an acute event [40]. In addition, older patients with heart failure are at particularly high risk of adverse drug reactions (ADRs), with prevalence ranging from 31% in patients at low frailty risk to 84% in those at high frailty risk [41]. In this context, drug interactions affecting chloride homeostasis represent a real and often underestimated clinical risk that may worsen prognosis and influence therapeutic management [42].

### 4.6. Diuretics

Among the medications most commonly prescribed in older adults (>80 years), and among the leading causes of iatrogenic hypochloremia, are loop diuretics, which are used by approximately 26% of the elderly population, with prevalence increasing with age [43]. Loop diuretics act through inhibition of the Na^+^-K^+^-2Cl^−^ cotransporter in the thick ascending limb of the loop of Henle, increasing chloride excretion up to twentyfold compared with baseline values [44]. Hypochloremia, in turn, contributes to diuretic resistance, leading to escalation of diuretic dosage and establishing a vicious cycle [45].

Thiazide diuretics, frequently used as antihypertensive agents, may also contribute to hypochloremia. By inhibiting the Na^+^-Cl^−^ cotransporter in the distal convoluted tubule, they increase chloride excretion, although to a lesser extent than loop diuretics but with a longer half-life [46].

### 4.7. Other Drugs Associated with Hypochloremia

Laxatives are widely used in older adults for the management of constipation, and their abuse may result in chloride loss through gastrointestinal depletion [47]. Excessive use of sodium bicarbonate may also lead to hypochloremia through the development of metabolic alkalosis [48].

Another class of medications widely prescribed in the elderly population is corticosteroids. In particular, corticosteroids with significant mineralocorticoid activity may stimulate mineralocorticoid receptors (MR) in the distal renal tubule and collecting duct, promoting sodium reabsorption along with potassium and hydrogen ion excretion. This mechanism leads to hypokalemic metabolic alkalosis, which is typically accompanied by secondary hypochloremia [49,50].

Selective serotonin reuptake inhibitors (SSRIs), commonly prescribed for depressive symptoms in older adults, are a well-known cause of Syndrome of Inappropriate Antidiuretic Hormone Secretion (SIADH). Increased vasopressin (ADH) release, acting on V2 receptors in the renal collecting ducts and promoting aquaporin-2 expression, results in excessive free water reabsorption and consequently dilutional hyponatremia and hypochloremia [51]. Several other drug classes are also implicated in the development of SIADH and may therefore contribute to hypochloremia through the same mechanism.

## 5. Epidemiology of Hypocloremia in Heart Failure

In recent years, growing interest in chloride has led to the availability of more robust epidemiological data. Hypochloremia is generally defined as a serum chloride concentration below 98 mEq/L, although threshold values vary among studies. In a recent systematic review and meta-analysis, the pooled prevalence of hypochloremia among patients with HF was estimated at 14% [52]. Other studies have reported prevalence rates ranging from approximately 10% to 33%, depending on patient characteristics and clinical setting, as shown in Table 1 [23,53,54,55,56,57,58,59,60,61,62,63,64,65,66].

Prevalence of hypochloremia in HF is substantially higher than that reported in the general population (3.8%) [67], suggesting that HF represents a clinical condition particularly prone to chloride dysregulation.

Notably, the prevalence of hypochloremia appears to increase progressively with HF severity and acuity, ranging from approximately 5.5% in stable chronic HF outpatients to 11% in worsening HF outpatients, and up to 24% in patients hospitalized for acute HF [64]. This epidemiological gradient is particularly relevant in older adults, who represent the majority of patients hospitalized for acute HF and present reduced renal reserve, greater exposure to diuretic therapy, multimorbidity, frailty, and polypharmacy. These factors increase susceptibility to both dilutional and depletional electrolyte disturbances, making hypochloremia especially frequent and clinically relevant in the geriatric population.

Despite the high burden of HF in older adults, data on the epidemiology of hypochloremia in this population remain scarce. In a large cohort of 1819 patients aged ≥60 years hospitalized with acute HF, hypochloremia was present in 25.0% of patients; notably, 40.8% of the study population was aged ≥80 years [58]. Furthermore, the only study specifically investigating very old patients hospitalized with acute HF reported a prevalence of 29% [23], suggesting that hypochloremia is highly prevalent in the oldest-old population. Nevertheless, its determinants and prognostic implications in this population remain insufficiently characterized.

## 6. Chloride and Heart Failure: The Patho-Physiological Mechanisms

Hypochloremia is a very common condition in patients with heart failure. Its pathogenesis is multifactorial [23] and includes the chronic use of loop diuretics, which promote urinary chloride loss, as well as neurohormonal activation (RAAS and ADH) and fluid retention leading to dilutional effects. Importantly, hypochloremia is a marker of disease severity and an independent predictor of poor prognosis in this population.

A reduction in chloride delivery to the distal nephron, particularly at the level of the macula densa, promotes activation of the RAAS [46]. This neurohormonal response leads to vasoconstriction, sodium and water retention, and ultimately contributes to the progression of heart failure. In parallel, loop diuretics inhibit the Na^+^-K^+^-2Cl^−^ cotransporter (NKCC2) in the thick ascending limb, causing significant chloride depletion. This further enhances RAAS activation and increases distal sodium reabsorption, promoting the development of diuretic resistance.

Moreover, hypochloremia is closely associated with metabolic alkalosis due to the reciprocal relationship between chloride and bicarbonate. Metabolic alkalosis, in turn, reduces the efficacy of diuretics and favors additional sodium reabsorption, further exacerbating fluid overload.

The overall result is the establishment of a vicious cycle in which chloride depletion, neurohormonal activation, and impaired diuretic responsiveness reinforce each other. This cycle contributes to worsening cardiac and renal function within the context of the cardiorenal syndrome and leads to persistent or worsening congestion.

## 7. Prognostic Role of Hypocloremia in Heart Failure

Hyponatremia has long been recognized as a well-established and robust marker of poor prognosis in patients with HF [6]. In recent years, however, increasing attention has been directed toward chloride, and a growing body of evidence has investigated the prognostic significance of hypochloremia in HF, as summarized in Table 2 [9,23,53,54,55,56,57,58,59,60,61,62,63,65,66,68,69,70,71,72].

In a retrospective analysis of 2699 patients with chronic heart failure (CHF) enrolled in the Beta-Blocker Evaluation of Survival Trial (BEST), Testani et al. demonstrated that when both sodium and chloride were included in a multivariable model, only chloride retained an independent association with 2-year mortality, whereas sodium lost its prognostic significance [54]. It should be noted that the BEST trial exclusively enrolled patients with chronic HF with reduced ejection fraction (HFrEF) receiving a non-selective beta-blocker; therefore, the generalizability of these findings to other HF phenotypes, particularly HF with preserved ejection fraction (HFpEF), remains uncertain. Similar results were subsequently reported by Grodin et al. in ambulatory patients with CHF followed for up to five years, further supporting the independent prognostic value of chloride [53]. Moreover, hypochloremia developing during follow-up has also been associated with an increased risk of mortality, suggesting that chloride abnormalities may reflect dynamic changes in disease severity and treatment response [56].

However, the prognostic superiority of hypochloremia over hyponatremia has not been consistently demonstrated across all clinical settings. Ferreira et al. reported that among patients with acute myocardial infarction complicated by systolic dysfunction and HF, low chloride concentrations were associated with increased mortality only in the presence of concomitant hyponatremia [73]. A similar interaction was observed by Zhang et al. in patients with CHF, where hypochloremia was associated with increased long-term mortality only when accompanied by low sodium levels [74].

Taken together, these findings suggest that chloride may provide prognostic information complementary to, rather than replacing, that offered by sodium. This concept is further supported by studies evaluating the sodium-to-chloride (Na:Cl) ratio [58,75], which integrates information from both electrolytes. In patients hospitalized for acute HF, an elevated Na/Cl ratio has been independently associated with increased all-cause mortality, highlighting the potential value of combined electrolyte assessment for risk stratification [75].

Evidence regarding the prognostic significance of hypochloremia in older adults with HF remains limited. In one of the few studies specifically focusing on this population, serum chloride—but not sodium—was associated with mortality and HF readmission according to a U-shaped relationship [23]. Notably, a depletional phenotype characterized by hypochloremia and low estimated plasma volume status was associated with significantly increased mortality compared with normochloremia, whereas dilutional hypochloremia accompanied by elevated plasma volume status was not independently associated with mortality. These findings suggest that, in older patients, the prognostic implications of hypochloremia may depend not only on chloride concentration itself but also on the underlying volume status and pathophysiological phenotype.

Additional evidence from elderly HF cohorts further supports the clinical relevance of chloride-based risk stratification. In a retrospective study including 1819 patients aged over 60 years hospitalized for acute HF, Fu et al. observed a U-shaped association between the Na/Cl ratio and 3-month mortality, with both low and high ratios associated with increased risk of death [58]. Similarly, in older patients with non-ischaemic dilated cardiomyopathy, low serum chloride concentrations were independently associated with both in-hospital and long-term adverse outcomes [76]. Overall, these findings indicate that hypochloremia is not only more prevalent in older adults and acute HF settings but may also identify distinct high-risk geriatric phenotypes in whom congestion, renal dysfunction, frailty, and diuretic-induced chloride depletion interact to worsen prognosis.

## 8. Phenotypes of Hypocloremia in Heart Failure

Recognizing distinct phenotypes of hypochloremia in heart failure is essential for understanding underlying pathogenetic mechanisms and informing therapeutic strategies. Recent studies have identified two principal forms: dilutional and depletive [77]. The dilutional phenotype involves a relative excess of free water compared to total body chloride, accompanied by pronounced neurohumoral activation, particularly of the RAAS and the arginine-vasopressin (AVP) axis. This activation results in increased reabsorption of free water and decreased serum sodium and chloride concentrations. In this context, hypochloremia arises from excessive dilution rather than an absolute deficit. Elevated concentrations of NT-proBNP and CA125, as well as clinical signs of hypervolemia, are often observed [78]. Conversely, the depletive phenotype is defined by an absolute reduction in chloride, typically resulting from diuretic therapy. Loop diuretics inhibit the NKCC2 cotransporter, leading to urinary chloride loss that exceeds urinary sodium loss. When this loss surpasses the body’s compensatory capacity, hypochloremia develops. This phenotype is associated with normal or increased serum sodium and hypochloremic metabolic alkalosis due to compensatory bicarbonate accumulation.

Although the literature frequently distinguishes between dilutional and depletive forms, in advanced heart failure, hypochloremia often results from multiple overlapping mechanisms. Residual congestion promotes arginine-vasopressin-mediated hemodilution, while extended administration of high-dose loop diuretics simultaneously induces absolute chloride depletion [11].

The differentiation between the underlying mechanisms may be aided by assessing the plasma sodium-to-chloride ratio, the urinary chloride-to-sodium ratio, fractional chloride excretion, and estimated plasma volume status (ePVS) [79,80].

Few studies have been conducted specifically on the elderly population, but it has been shown that the depletion phenotype (characterized by hypochloremia and low ePVS) was associated with a higher risk of mortality compared to normochloremic patients, while the dilution phenotype (hypochloremia and high ePVS levels) was not associated with increased mortality [23]. As suggested by the authors, this distinction may have an impact on the therapeutic approach by favoring the use of drugs that allow the shift of fluids from the extravascular compartment to the intravascular compartment (tolvaptan and SGLT2 inhibitors) and reducing the use of loop diuretics in patients with hypochloremia, hemoconcentration, and tissue congestion.

## 9. Potential Therapeutic Implications for Older Adults and Future Directions

The growing recognition of chloride as an active regulator of renal sodium handling, neurohormonal activation, and diuretic responsiveness has progressively shifted interest toward chloride-centered therapeutic strategies in HF. This concept may be particularly relevant in older adults, who are characterized by reduced renal reserve, frailty, multimorbidity, polypharmacy, and an increased susceptibility to both congestion and electrolyte disturbances.

Although current evidence does not support routine correction of hypochloremia as a therapeutic target per se, assessment of serum chloride may provide clinically relevant information to guide decongestion strategies, identify patients at increased risk of diuretic resistance, and support a more individualized therapeutic approach [79]. Importantly, however, most available evidence derives from studies conducted in the general HF population, whereas data specifically addressing older adults remain limited.

In particular, distinguishing between dilutional and depletional forms of hypochloremia may have important therapeutic implications in geriatric patients [23]. Dilutional hypochloremia is mainly associated with congestion and neurohormonal activation and may benefit from strategies promoting effective decongestion. In contrast, depletional hypochloremia is more frequently related to excessive chloride losses, metabolic alkalosis, reduced physiological reserve, and prolonged exposure to loop diuretics. In these patients, approaches aimed at preserving or restoring chloride balance may theoretically improve diuretic responsiveness and reduce the risk of further electrolyte disturbances.

Several therapeutic approaches, including acetazolamide, SGLT2 inhibitors, hypertonic saline solutions, and individualized diuretic regimens, have been proposed as potential chloride-sparing or chloride-restoring strategies. However, current evidence supporting their use specifically in older adults remains limited, and no randomized trials have evaluated a chloride-guided therapeutic strategy in this population. Consequently, there is currently insufficient evidence to support evidence-based recommendations for chloride-guided management in older patients with HF. Rather, these approaches should be considered promising but still investigational therapeutic perspectives that warrant further evaluation. Further prospective studies are needed to establish whether chloride-guided management can improve clinical outcomes, reduce rehospitalizations, and preserve functional status and quality of life in older adults with HF.

### 9.1. Acetazolamide

Acetazolamide is a carbonic anhydrase inhibitor that acts in the proximal tubule by reducing sodium and bicarbonate reabsorption. This mechanism promotes natriuresis, corrects metabolic alkalosis, and tends to preserve or increase serum chloride concentrations, potentially counteracting diuretic resistance [81,82].

The ADVOR trial, whose participants had a mean age of approximately 78 years, demonstrated that the addition of intravenous acetazolamide (500 mg daily) to standard loop diuretic therapy increased the likelihood of achieving successful decongestion within 72 h by 46%, without significant safety concerns [61]. These findings are particularly relevant for older adults, in whom metabolic alkalosis, diuretic resistance, and recurrent congestion frequently coexist.

However, the primary endpoint of ADVOR was short-term decongestion, and the study was not powered to detect differences in mortality, rehospitalization, or major cardiovascular outcomes. Furthermore, it was conducted before the widespread adoption of SGLT2 inhibitors, partially limiting its applicability to contemporary HF management. To date, no studies have specifically evaluated the role of acetazolamide as a chloride-guided strategy in frail older adults. Therefore, further prospective studies are warranted to establish its efficacy and safety in this particularly vulnerable population.

### 9.2. SGLT2 Inhibitors

SGLT2 inhibitors (empagliflozin and dapagliflozin), which are currently a cornerstone of therapy for both heart failure with reduced and preserved ejection fraction, promote natriuresis and osmotic diuresis through inhibition of sodium-glucose reabsorption in the proximal tubule. Unlike loop diuretics, they do not induce substantial chloride depletion or hypochloremic metabolic alkalosis, making them attractive candidates within a chloride-sparing therapeutic strategy [83].

Beyond their well-established benefits in reducing HF hospitalizations and cardiovascular mortality, SGLT2 inhibitors may facilitate effective decongestion while preserving electrolyte homeostasis and limiting neurohormonal activation. Their mechanism of action, characterized by a more physiological modulation of sodium and water balance, may be particularly advantageous in patients with hypochloremia and diuretic resistance.

Importantly, SGLT2 inhibitors have consistently demonstrated a favorable safety and tolerability profile across different age groups, including older and very old patients with frailty, multimorbidity, and chronic kidney disease [84]. In addition to their cardiovascular and renal benefits, emerging evidence suggests potential favorable effects on metabolic health, functional status, and cognitive outcomes, all of which are highly relevant therapeutic targets in the geriatric population [85]. These pleiotropic effects, together with their low propensity to induce electrolyte disturbances, make SGLT2 inhibitors particularly attractive in older adults with HF, in whom preservation of functional independence and quality of life are key treatment goals.

Nevertheless, the potential benefits of SGLT2 inhibitors specifically in hypochloremic HF patients remain largely speculative, and no studies have directly evaluated their efficacy according to chloride status or hypochloremic phenotype. Further research is needed to determine whether preservation of chloride homeostasis contributes to the clinical benefits observed with these agents and whether serum chloride may help identify older patients most likely to benefit from this therapeutic approach.

### 9.3. Hypertonic Saline Solution (HSS)

The administration of hypertonic saline solution in combination with loop diuretics has been suggested as a possible therapeutic option in patients with congestive HF and an inadequate response to conventional diuretic therapy. Beyond increasing plasma osmolality and promoting intravascular fluid recruitment, hypertonic saline may contribute to chloride repletion, thereby potentially improving diuretic responsiveness and mitigating neurohormonal activation [11,86].

Although several studies have reported improvements in congestion and diuretic efficiency, the available evidence remains limited and heterogeneous, and further studies are needed to define its efficacy and safety. Consequently, current guideline recommendations remain cautious, and routine use of hypertonic saline cannot be recommended.

Particular caution is warranted in older adults, given their increased vulnerability to rapid volume shifts, renal dysfunction, and treatment-related complications. Further randomized studies are needed to establish the efficacy and safety of this strategy in geriatric HF populations.

### 9.4. Personalized Therapeutic Strategy Based on Chloride Phenotype

Although current HF guidelines do not specifically recommend a chloride-guided therapeutic approach, emerging evidence suggests that serum chloride assessment may contribute to a more individualized management of congestion and diuretic resistance. In clinical practice, serum chloride should be routinely assessed at hospital admission for acute HF, before and during intensification of diuretic therapy, and during follow-up in patients with recurrent congestion, worsening renal function, or suspected diuretic resistance. Serial chloride measurements, interpreted together with bicarbonate, renal function, and volume status, may provide additional information beyond serum sodium levels alone.

Recognition of distinct hypochloremic phenotypes may be particularly relevant in older adults. In patients with dilutional hypochloremia, characterized by congestion and neurohormonal activation, therapies aimed at effective decongestion may be preferable. Conversely, in patients with depletional hypochloremia, often associated with prolonged loop diuretic exposure, metabolic alkalosis, and reduced physiological reserve, strategies aimed at preserving or restoring chloride balance may be more appropriate [23].

In this context, sequential nephron blockade with thiazide diuretics or metolazone may be considered in selected patients with persistent congestion and normal chloride concentrations. However, in the presence of hypochloremia, particularly when accompanied by metabolic alkalosis, chloride-sparing approaches such as acetazolamide and SGLT2 inhibitors may represent more physiologically appropriate options [11].

In frail older adults, clinicians should avoid indiscriminate escalation of loop diuretics or repeated sequential nephron blockade without reassessing chloride status, acid-base balance, renal function, and overall clinical context, as these interventions may exacerbate electrolyte disturbances and intravascular volume depletion.

At present, the available evidence does not yet allow evidence-based recommendations for a chloride-guided therapeutic approach. Therefore, the considerations presented above are intended to provide a conceptual framework for individualized clinical decision-making and should be viewed as emerging therapeutic perspectives rather than established treatment recommendations.

Prospective studies are needed to determine whether phenotypic classification of hypochloremia can improve clinical outcomes, reduce hospitalizations, and preserve functional status and quality of life in this vulnerable population.

## 10. Conclusions

Although only a few studies specifically investigating the prognostic role of hypochloremia in older adults with HF exist, current data suggest that low serum chloride levels are associated with adverse clinical outcomes and poorer prognosis also in this vulnerable population.

Therefore, routine assessment of serum chloride should be incorporated into the comprehensive evaluation of older patients with HF, alongside traditional clinical and laboratory markers. Emerging evidence also suggests that distinguishing between dilutional and depletional phenotypes of hypochloremia may have important therapeutic implications and could support a more individualized approach to decongestion strategies.

Further studies specifically involving geriatric HF populations are needed to clarify the mechanisms linking hypochloremia to disease progression, validate chloride-guided therapeutic approaches, and determine whether correction or prevention of hypochloremia can improve clinical outcomes, reduce hospitalizations, and enhance quality of life in older adults.

## Figures and Tables

**Table 1:** Main studies investigating the prevalence of hypochloremia in patients with heart failure.

First Author	Study Population	Sample Size	Prevalence of Hypochloremia
Grodin, 2016 [53]	Ambulatory patients with stable chronic HF	1673	13.6%
Testani, 2016 [54]	Patients with chronic HF	2699	13.0%
Cuthbert, 2018 [55]	HF outpatients	4705	10.7%
Bellino, 2021 [56]	HF outpatients	506	10%
Cuthbert, 2022 [57]	Patients discharged after HF hospitalization	963	15% at hospital admission; 36% at discharge
**Fu, 2023** **[58]**	**Older (≥60 years) patients hospitalized for acute HF (of whom 40.8% ≥80 years)**	**1819**	**25%**
Kurashima, 2023 [59]	Patients hospitalized for acute HF	2798	10% on admission; 17% at discharge
**Llàcer, 2023** **[23]**	**Older patients hospitalized for acute HF**	**429**	**29%**
Tan, 2024 [60]	ICU patients with HF	9364	12.5%
Van den Eynde, 2024 [61]	Patients hospitalized for acute HF	519	15%
Misumi, 2025 [62]	Post-discharge patients with chronic HF	2496	12.6%
Chen, 2025 [52]	Systematic review and meta-analysis of 18 studies including patients with acute and chronic HF	29,807	14%
Núñez, 2025 [63]	Patients hospitalized for acute HF	665	15%
Llàcer, 2025 [64]	Multicenter registry including chronic HF, ambulatory worsening HF (WHF), and inpatient WHF	1605	5.5% (chronic HF); 11% (ambulatory WHF); 24% (inpatient WHF)
Crespo-Aznarez, 2025 [65]	Patients hospitalized for acute decompensated HF (tertiary hospital cohort)	638	15.7%
Gebrie, 2026 [66]	Patients hospitalized for acute HF	260	33.1%

Note: Studies specifically investigating older and very old populations are shown in bold. Abbreviations: HF, heart failure; WHF, worsening heart failure; ICU, intensive care unit.

**Table 2:** Main studies investigating the prognostic role of hypochloremia in heart failure.

First Author	Study Population	Sample Size	Age (Years)	Outcome	Main Findings
Grodin, 2015 [9]	Hospitalized patients with acute decompensated HF (ADHF)	1318	68.7	All-cause mortality	Hypochloremia independently associated with increased mortality.
Grodin, 2016 [53]	Ambulatory patients with stable chronic HF	1673	67.3	5-year all-cause mortality	Hypochloremia independently associated with increased mortality.
Testani, 2016 [54]	Patients with chronic HF	2699	60.2	All-cause mortality	Hypochloremia independently associated with increased mortality.
Radulović, 2016 [68]	Hospitalized patients with acute HF	152	77	In-hospital mortality	Hypochloremia predictive of increased mortality.
Ter Maaten, 2016 [10]	Patients hospitalized for acute HF	1960	70.1	Mortality and diuretic response	Hypochloremia associated with diuretic resistance and reduced survival.
Kondo, 2018 [69]	Patients hospitalized with ADHF	208	76.7	HF death	Hypochloremia predicted HF mortality.
Cuthbert, 2018 [55]	Chronic HF outpatients	4705	73	All-cause mortality	Hypochloremia independently associated with increased mortality.
Bellino, 2021 [56]	Chronic HF outpatients	506	62	All-cause mortality	Hypochloremia independently associated with increased mortality.
Cuthbert, 2022 [57]	Patients discharged after HF hospitalization	963	75	Readmission or death	Discharge hypochloremia associated with higher risk of readmission or death.
Seo, 2022 [70]	Patients hospitalized with ADHF-HFpEF	870	81	All-cause mortality	Discharge hypochloremia independently associated with increased mortality.
**Fu, 2023** **[58]**	**Older (≥60 years) patients hospitalized for acute HF**	**1819**	**≥60 (40.8% ≥80)**	**All-cause mortality**	**U-shaped association between Na/Cl ratio and mortality.**
Kurashima, 2023 [59]	Patients hospitalized for acute HF	2798	76	All-cause mortality	Persistent hypochloremia was associated with an increased risk of death.
**Llàcer, 2023** **[23]**	**Older patients hospitalized for acute HF**	**429**	**85**	**All-cause mortality and HF readmission**	**Chloremia was associated with the risk of death and HF readmission in a U-shaped pattern.**
Wu, 2023 [71]	Meta-analysis of 7 studies including patients with acute and chronic HF	6787	62–67	All-cause death	Hypochloremia independently associated with increased mortality; persistent hypochloremia associated with worse prognosis.
Tan, 2024 [60]	ICU patients with HF	9364	75.5	In-hospital mortality	Hypochloremia associated with increased mortality.
Stankowski, 2024 [72]	Meta-analysis of 15 studies including patients with acute and chronic HF	25,848	55–81	All-cause mortality	Hypochloremia associated with increased mortality.
Van den Eynde, 2024 [61]	Patients hospitalized for acute HF enrolled in the ADVOR trial	519	78	Treatment response and all-cause mortality	Hypochloremia associated with slower decongestion and increased mortality.
Misumi, 2025 [62]	Post-discharge patients with chronic HF	2496	78	1-year mortality	Discharge hypochloremia associated with increased mortality.
Núñez, 2025 [63]	Older patients hospitalized for acute HF	386	82	Diuretic response	Hypochloraemia was associated with a worse diuretic response.
Crespo-Aznarez, 2025 [65]	Patients hospitalized for acute decompensated HF (tertiary hospital cohort)	638	79.7	All-cause mortality and HF rehospitalization	Admission hypochloremia independently predicted adverse outcomes.
Gebrie, 2026 [66]	Patients hospitalized for acute HF	312	51.6	Mortality and adverse outcomes	Hypochloremia associated with worse outcomes and mortality.

Note: Studies specifically investigating older and very old populations are shown in bold. Abbreviations: HF, heart failure; ADHF, acute decompensated heart failure; HFpEF, heart failure with preserved ejection fraction; ICU, intensive care unit.

## Data Availability

No new data were created or analyzed in this study. Data sharing is not applicable to this article.
